# Hepatitis B virus induces sorafenib resistance in liver cancer via upregulation of cIAP2 expression

**DOI:** 10.1186/s13027-021-00359-2

**Published:** 2021-03-23

**Authors:** Shouhua Zhang, Nuoya Li, Yanling Sheng, Wen Chen, Qiangliang Ma, Xin Yu, Jianping Lian, Junquan Zeng, Yipeng Yang, Jinlong Yan

**Affiliations:** 1 Department of General Surgery, Second Affiliated Hospital of Nanchang University, 1 Minde Road, Nanchang, Jiangxi China; 2 Department of General Surgery, Jiangxi Provincial Children’s Hospital, Nanchang, China; 3 Department of Ultrasound, The Affiliated Hospital of Jiangxi University of Traditional Chinese Medicine, Nanchang, China; 4 Department of Surgery, Jiangxi Provincial Cancer Hospital, Nanchang, China; 5 Department of dermatology, Ili Kazakh Autonomous State Chinese Medicine Hospital, Xinjiang, Uygur Autonomous Region China; 6 Department of Oncology, The Affiliated Hospital of Jinggangshan University, Ji’an, China; 7 Department of General Surgery, Xinhua Hospital of Shanghai Jiao Tong University School of Medicine, 1665 Kongjiang Rd, Shanghai, China

**Keywords:** Hepatitis B virus, cIAP2, Sorafenib resistance, Liver cancer, Lamivudine

## Abstract

**Background:**

HBV promotes cell survival by upregulating the expression of the cellular inhibitor of apoptosis protein 2 (cIAP2), however whether it is involved in HBV-induced sorafenib resistance in liver cancer remains unclear.

**Methods:**

cIAP2 overexpression and knockdown was adopted to assess the involvement of cIAP2 in HBV-induced sorafenib resistance. Anti-HBV drug lamivudine and Akt inhibitor were used to investigate the impact of HBV replication on cIAP2 expression and sorafenib resistance. Xenotransplantation mouse model was used to confirm the data on cell lines in vitro.

**Results:**

Liver cancer cell line HepG2.215 showed increased cIAP2 expression and enhanced resistance to sorafenib. Upon sorafenib treatment, overexpression of cIAP2 in HepG2 lead to decreased cleaved caspase 3 level and increased cell viability, while knockdown of cIAP2 in HepG2.215 resulted in increased level of cleaved caspase 3 and decreased cell viability, suggesting the involvement of cIAP2 in HBV-induced sorafenib resistance. Furthermore, anti-HBV treatment reduced cIAP2 expression and partially restored sorafenib sensitivity in HepG2.215 cells. Xenotransplantation mouse model further confirmed that co-treatment with lamivudine and sorafenib could reduce sorafenib-resistant HepG2.215 tumor cell growth.

**Conclusion:**

cIAP2 is involved in HBV-induced sorafenib resistance in liver cancer and anti-HBV treatments reduce cIAP2 expression and partially restore sorafenib sensibility.

## Background

Liver cancer is one of the most prevalent cancers in the world and also a leading cause of cancer-related death worldwide, resulting in about 841,000 new cases and more than 780,000 deaths every year [1, 2]. Although there are various risk factors for liver cancer, chronic infections with hepatitis B virus (HBV) are estimated to be responsible for at about 50% of cases in the world [3, 4]. HBV infection is one of the most common infections worldwide, with approximately 3.5% (257 million) of the world’s population are chronically infected with HBV and 10–25% of the HBV carriers have the lifetime risk of dying of either liver cancer or cirrhosis [5].

Currently, surgical resection, liver transplantation and radiofrequency ablation are the major treatment modalities for early stage liver cancer, while treatment options for advanced disease are very limited because conventional systemic chemotherapy has little effect [6]. Unfortunately, patients are mostly diagnosed in the advanced stage, with the 5-year survival rate less than 20% [7]. Up to now, sorafenib, a multitarget tyrosine kinase inhibitor, is the first and one of the few US FDA approved chemotherapy agents for advanced liver cancer [8]. Although sorafenib showed improved overall survival of patients with advanced liver cancer, its therapeutic effects are modest and also affected by several factors like HBV infection [9]. Clinical trials have suggested that liver cancer patients with HBV infection do not respond well to sorafenib than HBV negative liver cancer patients, indicating HBV may be able to induce sorafenib resistance in liver cancer via certain not yet known mechanisms [10].

Our previous study has shown that HBV infection can significantly induce the expression of the cellular inhibitor of apoptosis protein 2 (cIAP2) in liver cells via PI3K/Akt/NF-κB signaling pathway [11]. This finding implies that HBV promotes liver carcinogenesis through the modulation of cIAP2 expression. Since cIAP2 is a protein that supports cell survival by inhibiting cell apoptosis, whether it is involved in HBV-induced sorafenib resistance in liver cancer remains to be clarified. In this study, we aimed to investigate this question and also the possible underlying mechanism and potential strategies to overcome HBV-associated sorafenib resistance in liver cancer.

## Materials and methods

### Animals and ethical statement

Female BALB/c nude mice aging from 4 to 5 weeks were purchased from Beijing HFK Biotechnology and housed in specific pathogen-free condition with sterilized food, water and bedding provided. All experimental protocols involving animals were reviewed and approved by the Ethical Review Committee of Second Affiliated Hospital of Nanchang University (Approval Number: 2018083) and performed in accordance with the local regulations.

### Plasmids and cell lines

The following plasmids were used in this study. Mammalian expression vector pcDNA3.1(+) was purchased from ThermoScientific. To construct the cIAP2-expressing plasmid (p-cIAP2), cIAP2 coding sequence was obtained from Genebank (Accession number: NM_001165.5) and synthesized by GenScript Biotech and subcloned into pcDNA3.1(+).

Non-HBV expressing human liver cancer cell line HepG2 and persistent HBV-expressing liver cancer cell line HepG2.215 were both purchased from the American Type Culture Collection and cultured in DMEM with 10% FBS and antibiotics.

### Cell transfection and drug treatments

For plasmid transfection, HepG2 cells preseeded in 12 well plates were either untransfected or transfected with empty vector pcDNA3.1(+) or p-cIAP2, using Lipofectamine 2000 (Thermo Scientific) according to the manufacturer’s instructions. For siRNA transfection, HepG2.215 cells preseeded in 12 well plates were either untransfected, or transfected with control siRNA (4,390,844, Thermo Scientific) or cIAP2 siRNA (4,392,420, Thermo Scientific) both at the final concentration of 10 nM, using MISSION siRNA Transfection Reagent (Sigma-Aldrich) according the manufacturer’s instructions. Cells were either further treated with sorafenib or harvested as follows. For western blot analysis, cells were harvested 48 h post transfection, while for sorafenib treatment and cell viability assay, sorafenib at a final concentration of 7.5 μM was added 24 h post transfection and cell viability was assessed 48 h post sorafenib addition. For cells receiving lamivudine treatment, lamivudine at the final concentration of 100 μg/ml was added to cell culture for one week before cell seeding into plates.

### Cell viability assay

Cell viability was assessed using the MTT Cell Proliferation/Viability Assay Kit (R&D systems) according to the manufacturer’s instructions. In brief, MTT reagent at the dilution of 1:10 was added into cell culture and incubated for 4 h when purple precipitate was visible under the microscope. Then equal volume of Detergent Reagent was added to the medium and the plate was incubated for another 4 h in the dark. After incubation, Absorbance was measured with the testing wavelength of 570 nm and the reference wavelength at 650 nm. Cell viability was calculated with untreated cells being considered as 100% viable.

### Western blot

Western blot was conducted as previously described with modifications [12]. In brief, cells were harvested and lysed using Pierce™ IP Lysis Buffer (Thermo Scientific) supplemented with protease inhibitor cocktail (Roche). Cell lysate was then centrifuged at 10,000 g for 10 min to remove insoluble. Cleared cell lysate supernatant was resolved by a 4–12% SDS-PAGE gel and transferred onto a PVDF membrane. After transfer, the membrane was sequentially blocked with 5% nonfat milk for 1 h at room temperature, incubated with primary antibodies overnight at 4 °C, and with HRP-conjugated secondary antibodies for 1 h at room temperature. Three washes with PBST was done between incubations, and after the final wash, immuobands on the membrane was developed by ECL Plus Western Blotting Substrate (BosterBio) and imaged by ChemiDoc XRS+ System (Bio-Rad). Band intensity was quantified by Image J (version 1.53c, National Institute of Health, USA).

### Quantification of HBV DNA

Quantification of HBV DNA was performed as previously described with modifications [11]. In brief, DNA was extracted from cell culture supernatant using QIAamp DNA Mini Kit (Qiagen) and HBV DNA was quantified by TaqMan RT-PCR using SsoAdvanced™ Universal Probes Supermix (Bio-Rad) on a Bio-Rad CFX96 platform. The following primers were used: forward primer: 5′-TTCCTCTCATCCTGCTGC-3′, reverse primer: 5′-ACAAACGGGCAACATACCTTG-3′, and TaqMan probe FAM-TATGCCTCATCTTCTTRTTGGTT. β-actin was used in our study as a normalization control for the quantification of HBV DNA. The following primers were used: forward primer: 5′- AGGTGAGGCTGCAAACAGCTA-3′, reverse primer: 5′- TGAATCCTTTTCTGAGGGATGAA-3′, and TaqMan probe FAM- TGCACATTGGCAACAGCCCCTG. The thermocycling program was set as follows: polymerase activation and DNA denaturation: 95 °C, 3 min; 40 cycles of 95 °C, 15 s (denaturation) and 60 °C, 15 s (annealing and extension).

### Tumor cell injection and drug treatments

Tumor development in nude mice was performed as previously described with modifications [13]. In brief, HepG2 and HepG2.215 cells were harvested and washed with PBS and then resuspended at 2 × 10^7^ cells/ml in PBS. Each mouse was received 300 μl cells subcutaneously at the left flank. Drug treatment started when tumor volume reached around 100 mm^3^. Both sorafenib (prepared in 0.4% DMSO in PBS) and lamivudine (prepared in PBS) were injected at the dose of 100 mg/kg intraperitoneally once a day for 15 consecutive days. For the animals missing one or both of the drugs, mock treatment with solvent (0.4% DMSO in PBS for sorafenib and PBS for lamivudine) in the same volume was injected.

### Measurement of tumor volume, tumor weight and animal weight

Tumor size was measured every 3 days and tumor volume was calculated using the formula: $V={\left( mean\ diameter\right)}^3\times \frac{\pi }{6},$ as previously described [14]. Animal weight was also measured every 3 days. Tumor weight was measured on the day of animal sacrifice.

### Statistical analysis

All statistical analyses in this study was performed using GraphPad Prism 8.3.0 (GraphPad) and all numerical data were presented as mean ± standard deviation (SD). For comparisons between two groups, Mann-Whitney test was used, whereas for comparisons among three or more groups, One-way ANOVA with Dunn’s multiple comparison test were adopted. For all the analyses, a *p* value less than 0.05 was considered significant.

## Results

### HBV infection induces resistance to sorafenib in liver cancer cells

HBV infection induces resistance to sorafenib in liver cancer, but the underlying mechanism remains unclear. Our previous study showed that HBV infection significantly induced the expression of an anti-apoptotic protein, cIAP2 [11]. Whether the upregulation of cIAP2 contributes to the sorafenib resistance in HBV-infected liver cancer remains to be investigated.

**Fig. 1:**
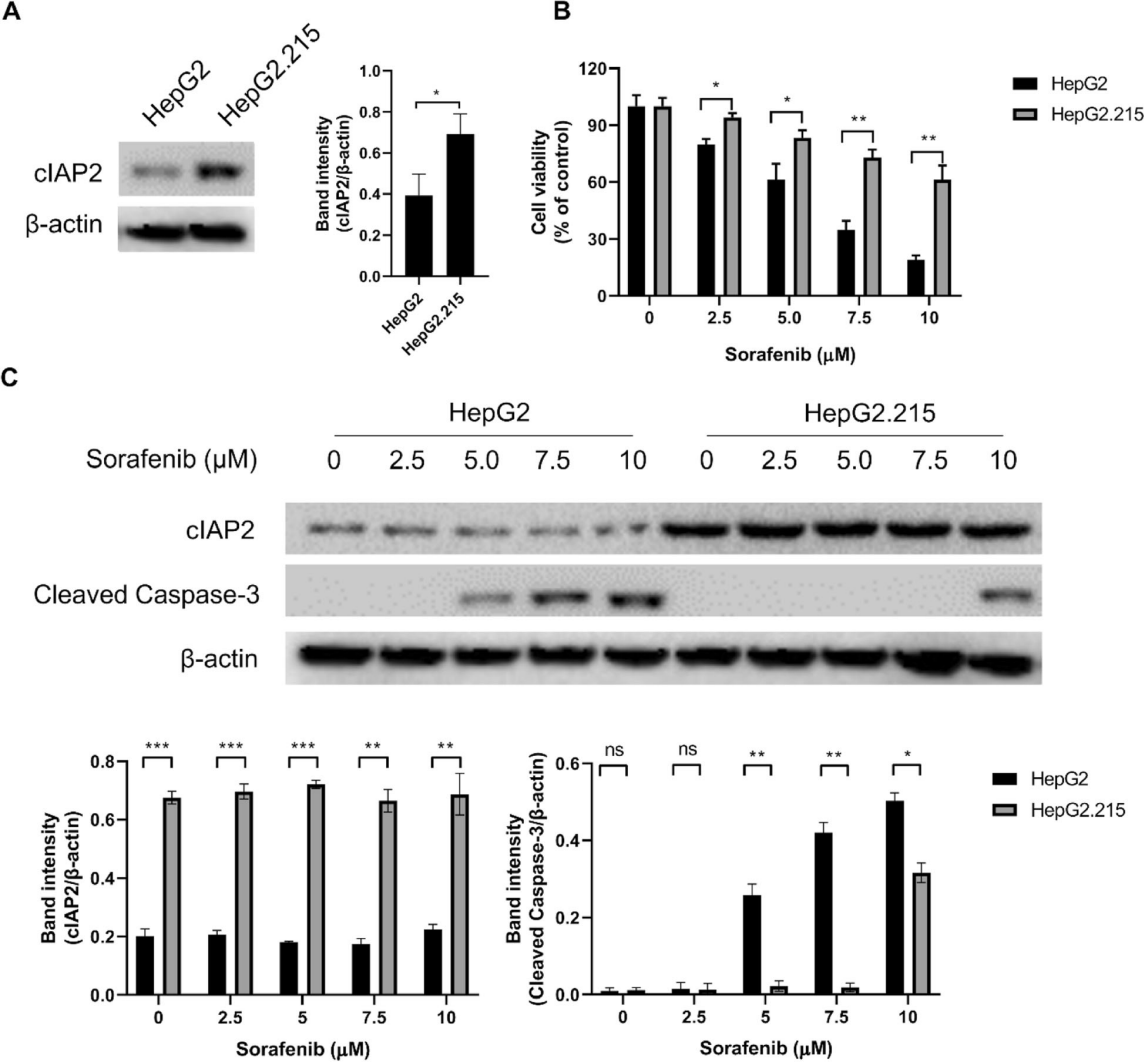
HBV infection induces resistance to sorafenib in liver cancer cells. **a** Liver cancer cell line HepG2 and HBV-transformed HepG2.215 cells were lysed and cIAP2 expression was determined by western blot. One representative data out of three is shown. **b** HepG2 and HepG2.215 cells were treated with ascendant doses of sorafenib for 24 h, and then cell viability was assessed by MTT. Data shown are mean ± SD of three independent experiments. *, *p* < 0.05; **, *p* < 0.01. **c** HepG2 and HepG2.215 cells were treated with ascendant doses of sorafenib for 24 h, and then cleaved caspase-3 was measured by western blot. One representative data out of three is shown

We used HBV-producing liver cancer line HepG2.215 and its parental non-HBV liver cancer cell line HepG2 as our in vitro models. First, we confirmed that cIAP2 expression was upregulated upon HBV infection in liver cancer cells (Fig. 1a). Next, we also tested the sensitivity of the two cell lines to sorafenib. Both HepG2 and Hep2.215 were treated with ascendant concentrations of sorafenib and the cell viability was assessed by MTT assay and cleaved caspase 3 expression. Our data showed that HepG2 was significantly more sensitive to sorafenib treatment than HepG2.215, as evidenced by MTT (Fig. 1b) and cleaved caspase 3 expression (Fig. 1c). In addition, the expression levels of cIAP2 in both HepG2 and HepG2.215 cells were determined after treatment with various doses of sorafenib and no apparent change was observed, indicating sorafenib treatment does not affect cIAP2 expression (Fig. 1c). Together, these data here indicate that HBV infection could upregulate cIAP2 expression, and also induce liver cancer cell tolerance to sorafenib.

### cIAP2 expression is associated with sorafenib resistance in liver cancer cells

**Fig. 2:**
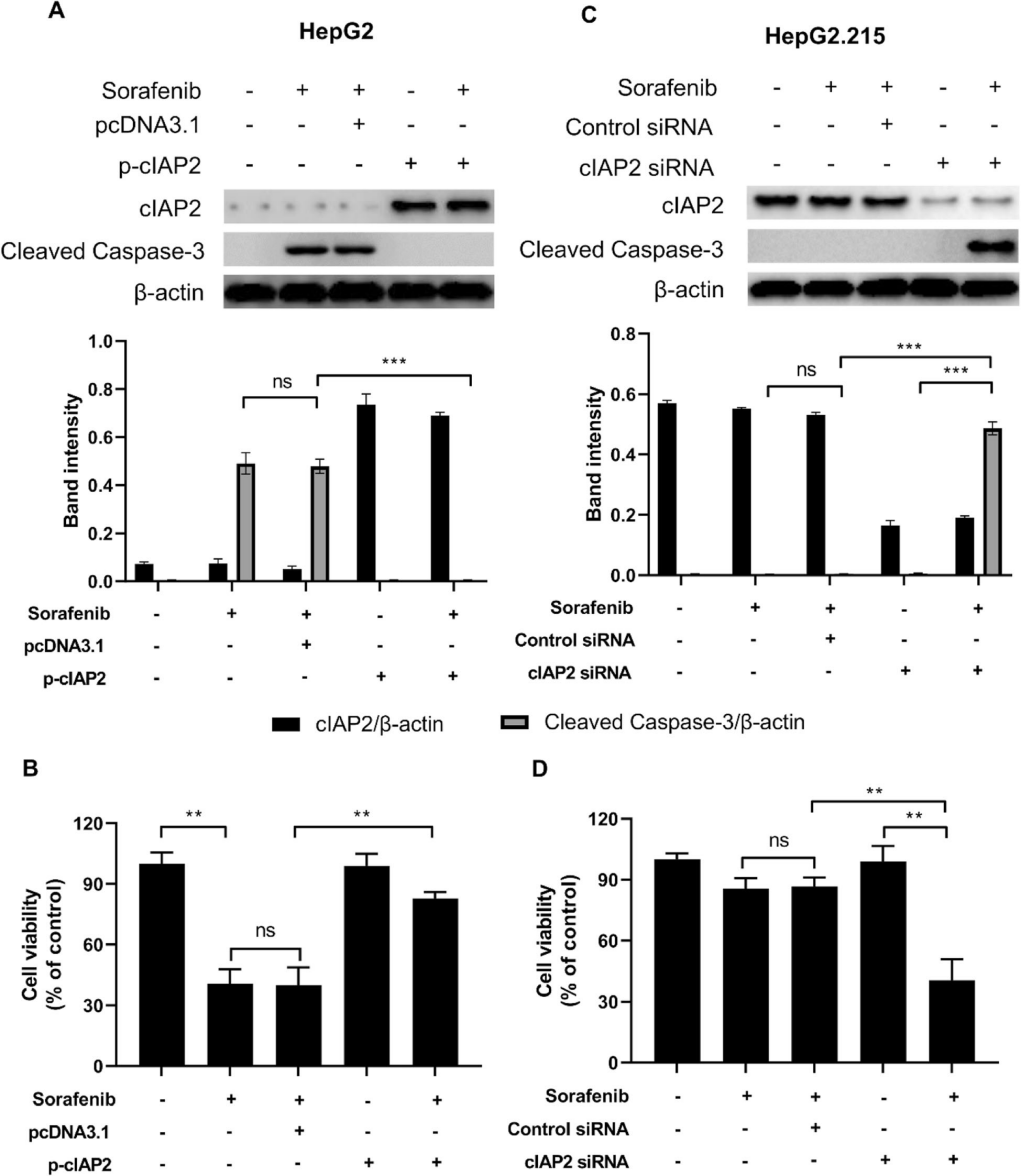
cIAP2 expression is associated with sorafenib resistance in liver cancer. **a-b** HepG2 cells were first transfected with pcDNA3.1 or p-cIAP2, and then treated with sorafenib. **c-d** HepG2.215 cells were first transfected with control siRNA or cIAP2 siRNA, and then treated with sorafenib. Twenty-four h later, **a** and **c** the expression of cIAP2 and cleaved caspase-3 were measured by western blot, **b** and **d** cell viability was determined by MTT. For western blot, one representative data out of three is shown. For cell viability, data shown are mean ± SD of three independent experiments. ns, not statistically significant; *, *p* < 0.05; **, *p* < 0.01

To further explore whether cIAP2 expression is associated with sorafenib resistance in liver cancer cells, HepG2 cells were transfected with p-cIAP2 to overexpress this protein and their sensitivity to sorafenib were assessed. As shown in Fig. 2a, when cIAP2 was overexpressed in HepG2 cells, the level of cleaved caspase 3 was considerably decreased. Similarly, sorafenib-induced cell death was significantly decreased in HepG2 cells with cIAP2 overexpression, comparing to those transfected with pcDNA3.1 (Fig. 2b). By contrast, HepG2.215 cells, when transfected with cIAP2 siRNA, showed higher level of cleaved caspase 3, comparing to cells transfected with control siRNA (Fig. 2c). Similarly, HepG2.215 cells with cIAP2 knockdown became significantly more sensitive to sorafenib, comparing to cells without cIAP2 knockdown (Fig. 2d). In addition, cIAP2 siRNA transfection only downregulated cIAP2 expression, without showing any apparent effect on the level of cleaved Caspase-3 and cell viability (Fig. 2c and d). Taken together, our data here indicate that cIAP2 expression is involved in the resistance of liver cancer cells to sorafenib.

### Anti-HBV treatment with lamivudine reduces cIAP2 expression and partially restores sensitivity to sorafenib in liver cancer cells

**Fig. 3:**
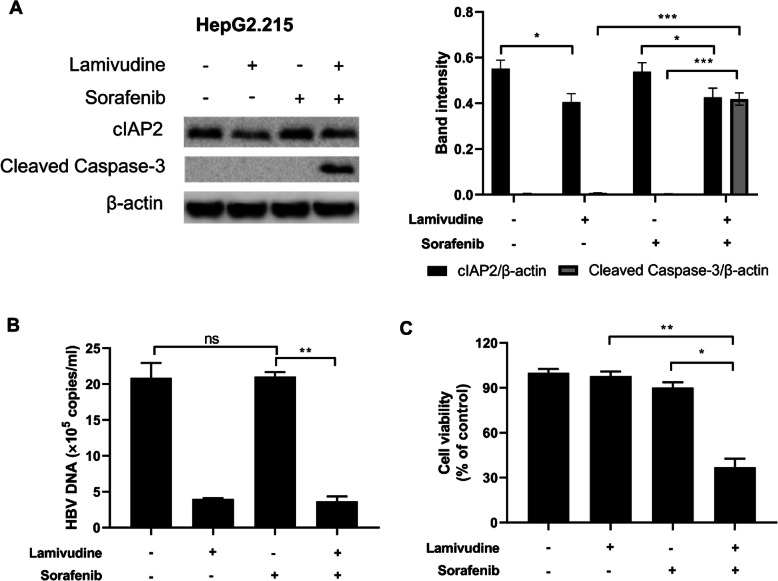
Anti-HBV treatment with lamivudine reduces cIAP2 expression and partially restores sensitivity to sorafenib in liver cancer cell. **a-c** HepG2.215 cells were first treated with or without lamivudine, and then further treated with sorafenib. Twenty-four h post sorafenib treatment, **a** the expression of cIAP2 and cleaved caspase-3 was determined by western blot, **b** HBV DNA in cultured supernatant was measured by RT-PCR, and **c** cell viability was assessed by MTT. For western blot, one representative data out of three is shown. For HBV DNA quantification and cell viability, data shown are mean ± SD of three independent experiments. ns, not statistically significant; *, *p* < 0.05; **, *p* < 0.01

Since HBV induces cIAP2 expression and cIAP2 expression is associated with sorafenib resistance in liver cancer cells, we further investigated whether inhibition of HBV replication would impact cell sensitivity to sorafenib. HepG2.215 cells were first treated with or without anti-HBV drug lamivudine, and then cell sensitivity to sorafenib was assessed. Treatment with lamivudine could reduce cIAP2 expression (Fig. 3a) and HBV replication (Fig. 3b), but did not induce cleaved caspase 3 expression (Fig. 3 a). Interestingly, after lamivudine treatment, cells became more sensitive to sorafenib, as evidenced by cleaved caspase 3 expression (Fig. 3a) and cell viability (Fig. 3c).

### Akt inhibitor treatment reduces cIAP2 expression and partially restores sorafenib sensitivity in liver cancer cells

**Fig. 4:**
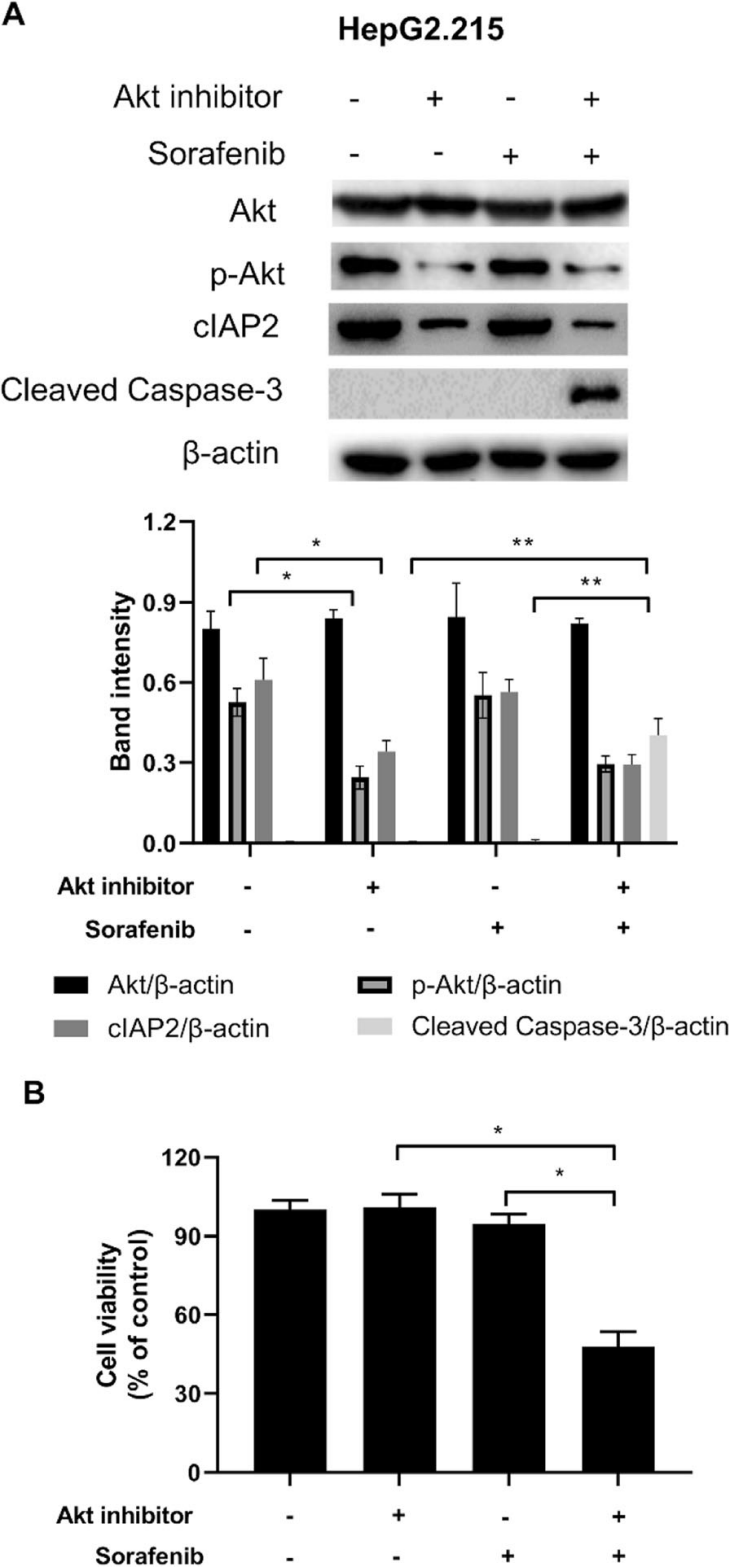
Akt inhibitor treatment reduces cIAP2 expression and partially restores sorafenib sensitivity in liver cancer cell. **a-b** HepG2.215 cells were first treated with or without Akt inhibitor MK-2206, and then further treated with sorafenib. Twenty-four h post sorafenib treatment, **a** the expression of cIAP2 and cleaved caspase-3 was determined by western blot, and **b** cell viability was assessed by MTT. For western blot, one representative data out of three is shown. For cell viability, data shown are mean ± SD of three independent experiments. ns, not statistically significant; *, *p* < 0.05; **, *p* < 0.01

Our previous study showed that HBV infection induced cIAP2 expression via PI3K/Akt/NF-κB signaling pathway and the inhibition of Akt could abort such cIAP2 enhancement [11]. Therefore, we here also investigated whether Akt inhibitor could affect the sensitivity of HBV+ liver cancer cells to sorafenib. HepG2.215 cells were first treated with Akt inhibitor (10 μM MK-2206) and/or sorafenib for 48 h, and then the expression of cleaved caspase 3 and cell viability was assessed. As shown in Fig. 4a, Akt inhibitor reduced the level of phosphorylated Akt (p-Akt) and the expression of cIAP2 in HepG2.215 cells. Furthermore, the combination of Akt inhibitor and sorafenib increased the level of cleaved caspase 3 (Fig. 4a). Similarly, the cell viability was also only reduced when cells were treated with the combination of Akt inhibitor and sorafenib (Fig. 4b). Taken together, these data here indicate that Akt inhibitor could partially restore sorafenib sensitivity in resistant liver cancer cells, probably through the downregulation of cIAP2 expression.

### Combination of lamivudine and sorafenib can reduce HBV+ liver tumor growth in mouse model

**Fig. 5:**
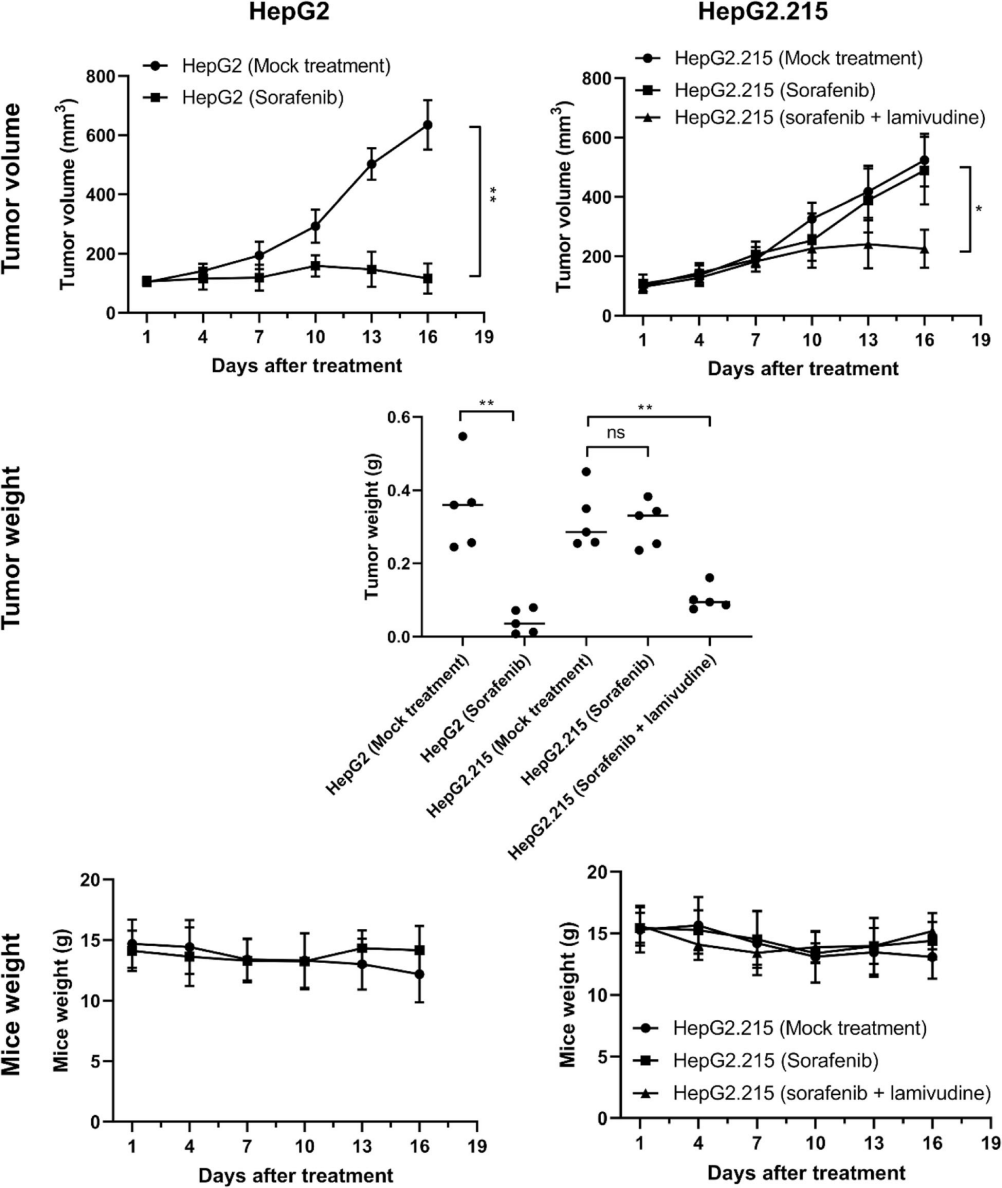
Combination of lamivudine and sorafenib can reduce HBV+ liver tumor growth in mouse model. Nude BALB/c mice were first injected with HepG2 or HepG2.215 cells to form tumors. Then mice were either mock treated with solvent, or treated with sorafenib, or sorafenib and lamivudine. Tumor volume and mice weight were measured every 3 days. Tumor weight was measured at the day of mice sacrifice. Data shown are mean ± SD of three independent experiments. ns, not statistically significant; *, *p* < 0.05; **, *p* < 0.01

**Fig. 6:**
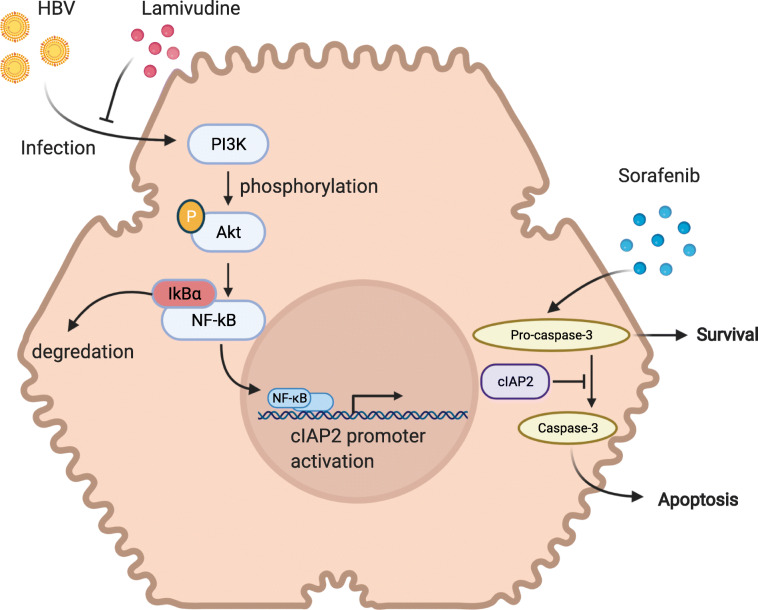
HBV induces sorafenib resistance in liver cancer via upregulation of cIAP2 expression. HBV infection induces cIAP2 expression through PI3K/Akt/NF-κB signaling pathway. The upregulated cIAP2 promotes cell survival by reducing caspase-3 level. The cIAP2-enhanced cell survival renders the cells more resistant to sorafenib treatment, while anti-HBV treatment with lamivudine can suppress the upregulation of cIAP2 and consequently restore cancer cell sensitivity to sorafenib

The in vivo efficacy of lamivudine in combination with sorafenib in suppressing liver cancer growth was also assessed in xenotransplantation mouse model. Similar to the data observed in cell lines, in HepG2-derived tumor, sorafenib treatment alone for two weeks significantly suppressed tumor growth, comparing to mock treatment group. However, in HBV+ HepG2.215-derived tumor, sorafenib alone did not shown apparent suppression in tumor volume reduction. Only did the combination of lamivudine and sorafenib significantly slowed down the tumor growth (Fig. 5, tumor volume and weight). During the treatment of sorafenib and/or lamivudine, mice body weight was also monitored, and no apparent changes were observed among different treatments (Fig. 5, mice weight). Together, the data here indicate that HBV+ HepG2.215 cells are resistant to sorafenib and the co-treatment with anti-HBV drug lamivudine can restore the sensitivity of HepG2.215 cells to sorafenib and consequently reduce tumor growth (Fig. 6).

## Discussion

With very limited treatment options, liver cancer, one of the most common cancers worldwide, is a big threat to the health of human and a huge economic burden to the society. The approved use of the first chemotherapeutic drug sorafenib has marked a new era in liver cancer treatment. Although sorafenib has improved overall survival of patients with advanced liver cancer, the tumor-response rate of sorafenib in liver cancer is low and also sorafenib resistance is observed, especially in HBV-infected patients. Therefore, to understand the mechanism underlying HBV-induced sorafenib resistance in liver cancer is important for the optimization of sorafenib efficacy and/or development of new treatment strategies in liver cancer. Based on our previous finding that HBV promotes liver cancer cell survival by upregulation of cIAP2 expression via PI3K/Akt/NF-κB signaling pathway, we, in the current study, further investigated whether HBV-induced cIAP2 expression was involved in sorafenib resistance in liver cancer. Our findings have shown that cIAP2 expression is involved in HBV-induced sorafenib resistance in liver cancer, and that the inhibition of HBV replication and cIAP2 upregulation Akt pathway could partially restore cancer cell sensitivity to sorafenib. This study provides not only a mechanism for the HBV-induced sorafenib resistance in liver cancer, but also a possible way to treat such condition.

Sorafenib, the so-far one of the few approved drug for patients with advanced liver cancer, is a multiple kinase inhibitor [15]. Sorafenib targets serine-threonine kinase Raf-1 and some other tyrosine kinases like vascular endothelial growth factor receptors 1, 2 and 3, platelet-derived growth factor receptor and fibroblast growth factor receptor [15, 16]. Although phase III clinical trial showed that sorafenib increased survival rate, the rate of patients responding to sorafenib is actually very low (2–3%), especially in HBV positive population [15–17]. Therefore, it is of great importance to understand the mechanism of sorafenib resistance, in order to develop effective strategies to cope these problems. A number of studies have shown that several pathways are involved in sorafenib resistance, including PI3K/Akt and JAK-STAT pathways, the activation of hypoxia-inducible pathways and the epithelial-mesenchymal transition [18–21]. However, the mechanism linking HBV infection and sorafenib resistance has not yet been clearly elucidated. Our previous study has shown that HBV infection could enhance the expression of cIAP2 via PI3K/Akt/NF-κB pathway in liver cancer [11]. In the current study, our findings have further shown that HBV-induced cIAP2 upregulation is involved in sorafenib resistance. Moreover, inhibition of Akt, downregulation of cIAP2 and suppression of HBV replication can partially restore the liver cancer cell sensitivity to sorafenib, further implying the involvement of cIAP2, Akt pathway and HBV infection in the development of sorafenib resistance.

cIAP2 is a member of the inhibitor of apoptosis protein family, which sustains cell survival by inhibition of caspase activity [22–24]. The upregulation of cIAP2 has been observed in many forms of cancers, including liver cancer [25–27]. Our study here has indicated that the enhancement of cIAP2 expression by HBV infection contributes to the development of sorafenib resistance. However, we have also observed that when cIAP2 expression was downregulated via various means including cIAP2 knockdown, inhibition of Akt and HBV replication, the sorafenib resistance has only been partially overcome, indicating that other factors may also be involved in sorafenib resistance in HBV infected liver cancer. According to previous publications, HBV can interfere cell viability in several ways. Directly, HBV encodes a few oncogenesis genes like HBx and preS2/S, which promote cell survival and induce carcinogenesis [28–32]. Indirectly, HBV can increase the expression of many anti-apoptotic host genes like BCL-2, survivin and XIAP, which again support cell survival and inhibit cell death [33, 34]. Although it is beyond the scope of our current study, it would be interesting to investigate whether these alterations by HBV infection contribute to sorafenib resistance in liver cancer.

## Conclusion

Taken together, our findings have shown that cIAP2 expression is involved in HBV-induced sorafenib resistance in liver cancer, and that the inhibition of HBV replication and cIAP2 upregulation Akt pathway could partially restore cancer cell sensitivity to sorafenib. This study provides not only a mechanism for the HBV-induced sorafenib resistance in liver cancer, but also a possible way to treat such condition.

## Data Availability

The raw data supporting the conclusions of this manuscript will be made available by the authors, without undue reservation, to any qualified researcher.

## Abbreviations

HBV: Hepatitis B virus
cIAP2: Cellular inhibitor of apoptosis protein 2
